# Conscious Tactile Perception Depends on Early Processing in Primary Somatosensory Cortex

**DOI:** 10.1111/ejn.70548

**Published:** 2026-05-15

**Authors:** Louisa Gwynne, Luigi Tamè

**Affiliations:** ^1^ School of Psychology University of Kent Canterbury UK

**Keywords:** conscious perception, somatosensory cortex, tactile detection, temporal processing, transcranial magnetic stimulation

## Abstract

The primary somatosensory cortex (S1) has long been implicated in tactile perception, yet its precise role in conscious tactile detection remains uncertain. The current study investigated the causal and time‐specific involvement of S1 in tactile detection using single‐pulse transcranial magnetic stimulation (spTMS). In two experiments, spTMS was applied over contralateral S1, an active control site (inferior parietal lobe; IPL), or under a sham condition at short (25 and 75 ms; Experiment 1) or longer (130 ms; Experiment 2) intervals following electrotactile stimulation of the finger. Participants performed a go/no‐go detection task at sensory threshold. In Experiment 1, tactile sensitivity was significantly reduced following early S1 stimulation compared with both active control and sham conditions. In contrast, no such effect was observed at a later timepoint in Experiment 2. Self‐reported TMS‐related distraction ratings did not account for the observed sensitivity differences, suggesting sensitivity‐specific modulation by early TMS rather than general task disruption. Together, these findings support a causal role for early S1 activity in conscious tactile detection. We propose that disruption at this early stage impairs initial encoding of tactile input, thereby attenuating subsequent perceptual awareness. Overall, the results underscore the critical contribution of S1 in conscious tactile detection and are compatible with recent accounts in which conscious tactile perception emerges from processing within a distributed neural network.

## Graphical abstract

Single‐pulse TMS over the contralateral primary somatosensory cortex (S1) impaired tactile sensitivity at early (25 and 75 ms), but not late (130 ms) latencies following electrotactile stimulation of the finger at the sensory detection threshold, demonstrating a temporally constrained role of S1 in conscious detection. TMS did not have any interference effect when it was delivered to an active control site or in a sham TMS condition (away from S1), confirming that the observed disruption is specific to S1. These findings show that S1 is critical for the initial encoding of tactile signals, serving as an early base for signal processing that underlies later higher‐order functions such as tactile retention and discrimination.

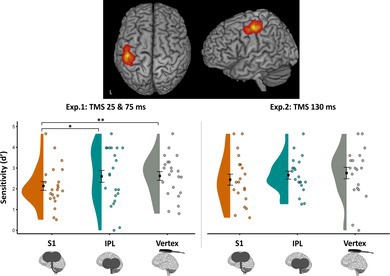

**Abbreviations**
BA: Brodmann area
EEG: electroencephalography
EHI: Edinburgh Handedness Inventory
EMG: electromyography
ERP: event‐related potential
FDI: first dorsal interosseous
IPL: inferior parietal lobe
ITI: intertrial interval
M1: primary motor cortex
mA: milliampere
MEP: motor‐evoked potential
MNI: Montreal Neurological Institute
ms: milliseconds
PPC: posterior parietal cortex
RMT: resting motor threshold
RT: reaction time
S1: primary somatosensory cortex
S2: secondary somatosensory cortex
SEP: somatosensory evoked potential
spTMS: single‐pulse transcranial magnetic stimulation
TMS: transcranial magnetic stimulation

## 1 Introduction

The ability to detect touch supports our fundamental need to interact with the external world. Early primate studies showed that the primary somatosensory cortex (S1) receives tactile input via thalamocortical projections to Brodmann areas (BA) 3b and 1 (Lamotte and Mountcastle 1979). These signals arrive approximately 20 milliseconds (ms) after contralateral median nerve stimulation and around 20–25 ms after digit nerve stimulation, marking the earliest cortical processing of tactile input (Allison et al. 1991; Peterson et al. 1995; Tsujinaka et al. 2023). Moreover, somatosensory‐evoked responses in S1 have been associated with perceptual detection (e.g., Jones et al. 2007; Palva et al. 2005). Nonetheless, the precise role of S1, encompassing the specifics of what it does and how it operates, remains open and disputed.

The earliest component of the somatosensory evoked potential (SEP) following peripheral stimulation is reflected in the N20 component in electroencephalography (EEG) or the M20 component in magnetoencephalography (MEG). This early negative potential is generated in BA 3b, contralateral to the locus of stimulation (Allison et al. 1991; Mauguière et al. 1997). Although the N20/M20 is widely considered the first cortical response to tactile input (Allison et al. 1991; Desmedt and Tomberg 1989; Peterson et al. 1995), its role in conscious detection remains debated. Seminal work by Libet and colleagues demonstrated that such early cortical responses do not reflect conscious awareness of the stimulus (Libet et al. 1967). In contrast, the slightly later P50 component—primarily originating from BAs 1 and 3b (Allison et al. 1992)—has been found to correlate with tactile detection (Soininen and Järvilehto 1983). Furthermore, M/EEG components peaking between 30 and 70 ms post‐stimulus have been shown to predict tactile detection performance in both non‐human primates (Cauller and Kulics 1991) and humans (Hirvonen and Palva 2016; Jones et al. 2007; Palva et al. 2005).

In contrast to the view that early components originating in S1 reflect stimulus detection, other research suggests these responses instead represent pre‐conscious sensory processing rather than perceptual awareness (Schubert et al. 2006; Wühle et al. 2010, 2011). In such a view, later SEP components occurring after 100 ms, such as the P100 and N140, are more consistently associated with conscious detection, reliably distinguishing between detected and undetected targets (Auksztulewicz et al. 2012; Forschack et al. 2020; Schröder et al. 2021; Schubert et al. 2006; Wühle et al. 2011). Both components have origins in the secondary somatosensory cortex (S2), with the P100 also originating in S1 (Allison et al. 1992; Mauguière et al. 1997). Taken together, this evidence highlights ongoing uncertainty regarding S1's contribution to detection, particularly the latency at which the tactile signal is key for conscious perception.

In addition to M/EEG research, several transcranial magnetic stimulation (TMS) studies have investigated the causal role of S1 in tactile detection (Cohen et al. 1991; Hannula et al. 2005; Harris et al. 2002; Seyal et al. 1997; Tamè and Holmes 2016). A seminal study by Cohen et al. (1991) demonstrated that single‐pulse TMS (spTMS) over the contralateral sensorimotor cortex suppressed electrotactile detection 200 ms before and 20 ms after stimulus onset (Cohen et al. 1991). Similarly, spTMS over contralateral S1 was found to impair the detection of electrotactile stimulus trains when administered 100 ms before and 20 ms after touch (McKay et al. 2003), suggesting a contribution of early S1 processing to tactile detection. However, later studies yielded inconsistent findings. For instance, spTMS over S1 failed to attenuate tactile detection, a result attributed to the absence of simultaneous motor cortex stimulation present in earlier studies (Koch et al. 2006). Nonetheless, magnetic resonance imaging (MRI)–navigated TMS applied to S1 at 20 or 50 ms post‐stimulus was shown to impair detection (Hannula et al. 2005), reinforcing the potential contribution of S1 involvement in conscious tactile detection.

In a recent TMS study, mechanical tactile detection was impaired by two successive spTMS pulses delivered over contralateral S1 25 and 75 ms after stimulus onset (Tamè and Holmes 2016). Notably, this effect was present only in a one‐interval ‘yes/no’ design task, but not in a two‐interval forced‐choice paradigm requiring the identification of a target in the first or second interval. This finding suggests that the contribution of S1 to tactile detection may depend on the cognitive demands of the task (Tamè and Holmes 2016; see also Katus et al. 2015). Moreover, the temporal window of S1 involvement in conscious detection may differ as a function of the stimulation type, namely, mechanical or electrical (Alouit et al. 2025). What is more, the temporal dynamics of its contribution to conscious detection remain largely unexplored.

Beyond detection, it is well‐evidenced that S1, in both monkeys and humans, contributes to later tactile processes of higher‐order phenomena, such as discrimination (Hannula et al. 2008; Harris et al. 2002; Kulics 1982; Kulics and Cauller 1986; Lamotte and Mountcastle 1979; Morley et al. 2007; Mountcastle et al. 1969; Romo et al. 2000), as well as interhemispheric integration (Tamè, Pavani, Papadelis, et al. 2015), retention (Harris et al. 2001, 2002; Katus et al. 2015), sensorimotor integration (Tamburin et al. 2001; Tamè, Pavani, Braun, et al. 2015) and localisation (Braun et al. 2005; Harris et al. 2001; Iwamura et al. 2002; Katus et al. 2015). Moreover, M/EEG studies tend to associate detection with later processing (beyond the N20); however, TMS evidence points to an earlier, causal role. This underscores the uncertainty surrounding the timing of S1's involvement in tactile detection and, most importantly, its key contribution to tactile awareness.

In the present study, we employed a spTMS paradigm in which TMS was delivered to contralateral S1, 25 and 75 ms (Experiment 1) or 130 ms (Experiment 2) after the onset of electrotactile stimulation at sensory threshold to the index finger during a go/no‐go task. The effect of spTMS over S1 was compared with an active control site (inferior parietal lobe; IPL) and a sham condition, in which the coil was flipped over the vertex. It was hypothesised that causal involvement of S1 to stimulus detection would be marked by significantly reduced stimulus sensitivity compared with both the active control and sham TMS conditions. In line with both TMS and M/EEG literature, we predicted significantly reduced tactile sensitivity in both Experiment 1 and 2 by TMS over S1.

## 2 Materials and Methods

### 2.1 Participants

An a priori power analysis using G*Power (Faul et al. 2007) estimated that a sample of 19 participants would be sufficient to achieve 80% (alpha = 0.05) to detect a medium‐to‐large effect (*dz* = 0.6) in a within‐subjects *t*‐test comparing tactile sensitivity (*d′*) between the experimental and control TMS sites. To mitigate for possible dropout and adequate performance on the task we aimed for a sample of 25 participants. In Experiment 1, three participants were excluded from analysis due to below‐chance performance in at least one of the TMS conditions, leaving a final sample of 22 participants (Mean_Age_ = 20.23, SD_Age_ = 5.28 years; 4 males, 18 females). Twenty participants were right‐handed and two left‐handed as indicated by self‐report on the Edinburgh Handedness Inventory questionnaire (EHI; Oldfield 1971; Mean = 72.4, range = −55.56 to +100). In Experiment 2, twenty‐two new participants were tested, matching the sample size of Experiment 1; however, two were removed for below‐chance performance in at least one of the TMS conditions, leaving a sample of 20 (Mean_Age_ = 22.9, SD_Age_ = 7.55 years; 5 males, 15 females). Nineteen were right‐handed and one left‐handed (EHI; M = 81.54, range = −75 to +100). All participants were recruited voluntarily in exchange for monetary payment at a rate of £7.50 an hour or via a university participation scheme for course credit. Participants had a normal sensation of the hands and arms as indicated through free self‐report. Participants were screened for TMS contraindications, with all reporting no neurological or psychiatric conditions and no current use of psychiatric and neuroactive medications. All procedures were approved by the University of Kent, School of Psychology ethics committee, and adhered to published TMS safety guidelines (Rossi et al. 2021).

### 2.2 Tactile Stimuli

Tactile stimulation consisted of a single transcutaneous electrical stimulus delivered through two stainless steel electrode rings, fitted at the proximal and intermediate phalange of the participants' right index finger, connected to a bipolar constant current stimulator (DS5; Digitimer, Welwyn Garden City, United Kingdom), controlled using a custom‐made programme in MATLAB (version 2019b) through a National Instruments data‐acquisition card (6001). Square wave pulse width was set at 0.2 ms. At low intensities (< ~6 mA), this created a singular, fast onset‐offset tap‐like sensation by momentary activation of predominantly Aβ mechanoreceptive fibres (Johnson 2007; Shiroshita et al. 2021). The right hand was always covered from sight throughout the experiment to avoid uncontrolled effects caused by vision of the body (Cardini et al. 2011; Tamè et al. 2017).

#### 2.2.1 Tactile Threshold

Tactile stimuli were delivered at the participants' electrotactile detection threshold, characterised as the minimum stimulator output detectable (Table 1). This was estimated with an automated 2‐down/1‐up staircase procedure converging to an approximately 71% perceivability (Levitt 1971). Starting at 0.1 mA, the stimulation increased by 0.3‐mA step sizes, reduced to 0.1 mA after the first reversal and to 0.02 mA after the second reversal. The staircase ended at the eighth reversal, and the sensory detection threshold (SDT) was calculated as the average of the last two reversals. This procedure was determined by a series of pre‐experiment pilots showing that this produced a stimulus 70%–80% perceivable on 10 out of 20 trials. Electrotactile detection thresholds did not differ between participants in the two experiments (*t* = 0.001, *p* = 0.999).

**TABLE 1:** Resting motor thresholds (RMT) and sensory detection thresholds (SDT) across experiments.

Participant	RMT (% MSO)		SDT (mA)	
	Exp 1.	Exp 2.	Exp 1.	Exp 2.
1	40.5	43.0	1.53	1.68
2	34.0	49.5	0.83	0.95
3	47.5	35.0	1.83	1.45
4	38.0	48.0	1.79	1.23
5	40.5	47.5	1.82	1.53
6	37.5	44.5	1.63	2.34
7	40.5	41.5	2.26	1.63
8	42.5	36.0	0.94	1.48
9	41.5	39.0	2.03	1.19
10	48.5	51.5	1.30	1.25
11	37.5	37.5	1.21	1.75
12	42.0	46.0	1.61	2.36
13	33.0	41.5	1.55	2.19
14	39.5	41.0	1.46	2.44
15	45.5	47.5	1.45	1.65
16	41.5	45.0	1.24	1.33
17	47.5	38.5	2.50	0.75
18	38.5	40.5	2.17	0.96
19	50.0	55.0	1.56	1.53
20	42.0	41.5	2.10	1.69
21	43.5	—	1.23	—
22	44.5	—	1.19	—
Mean	41.64	43.48	1.6	1.57
SD	(4.43)	(5.29)	(0.43)	(0.48)

Abbreviation: MSO = maximum stimulator output (%).

### 2.3 TMS and Neuronavigation

Biphasic spTMS was delivered with a MAG and MORE PowerMAG 100 Repetitive Magnetic Stimulator via a 100‐mm diameter figure‐of‐eight coil. Electromyography (EMG) was recorded with two Ag‐AgCI surface electrodes placed over the first dorsal interosseus (FDI) muscle of the right hand in a belly–tendon montage with a ~2‐cm inter‐electrode distance. The ground electrode was placed 1–2 cm proximal to the right pisiform bone of the left wrist, and skin preparation was completed using an alcohol swab. EMG signal was sampled in BrainSight Neuronavigation software (version 2.4.10; Rogue Research Inc., Montreal, QC) with a 3000‐Hz sampling frequency. Scalp localisation was optimised using the same BrainSight Neuronavigation software. The total number of TMS pulses delivered to each participant was between 220 and 480 (including M1 localisation and TMS threshold procedures).

#### 2.3.1 M1 Localisation and RMT

TMS intensity was delivered at 120% of the resting motor threshold (RMT), defined as the optimal scalp positioning to elicit at least a 50‐μV peak‐to‐peak motor‐evoked potential (MEP) in the right FDI muscle while at rest in five out of 10 trials (Rossini et al. 1994). A 4 × 4 square grid with 10‐mm spacing was drawn onto the participant's reconfigured digitalised scalp in BrainSight, centred on our lab average left M1‐FDI coordinates from previous experiments (Gwynne and Tamè 2025, bioRxiv). TMS was applied at 35% of the maximum stimulator output, increasing in increments of 5% until muscle‐evoked activity was visible on the EMG, and the coil was moved across the grid to find the grid point with the maximum MEP output. TMS intensity was then adjusted until desired MEPs were recorded. The coil handle was positioned 45° relative to the sagittal plane. Across both experiments, the mean RMT at the left M1 FDI hotspot (corresponding to the right FDI muscle) was 42.51% (SD = 4.89; Table 1), and the mean left M1‐FDI MNI coordinates were −37.76, −9.29, 59.06 (SD = 3.65, 6.35, 3.02; *x*, *y*, *z*).

#### 2.3.2 S1 Localisation

**FIGURE 1:**
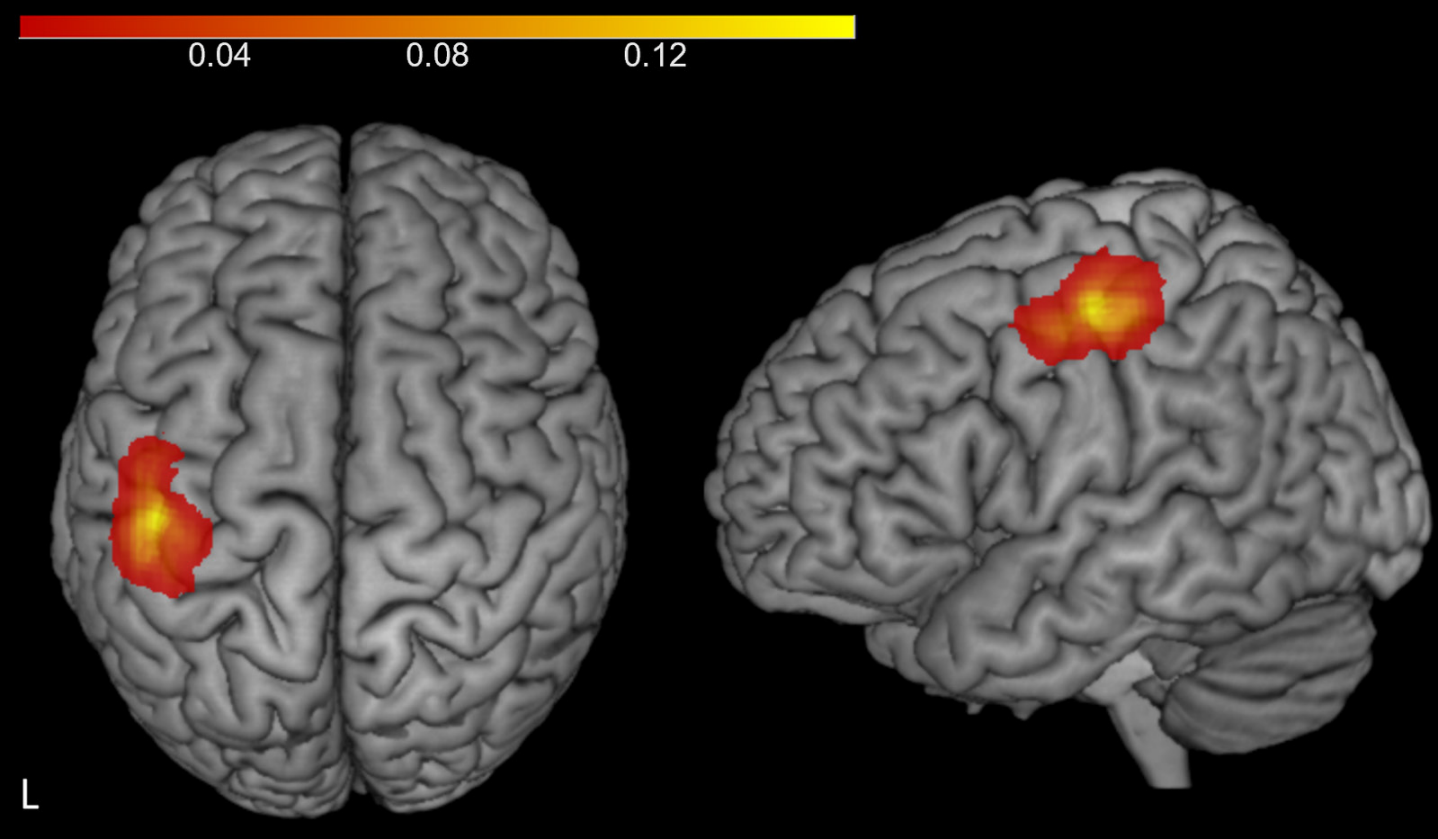
Showing determined left S1 MNI coordinates across participants and experiments. Heatmaps were made using custom MATLAB scripts and SPM12. Participants' MNI coordinates were transformed into voxel space of an anatomical template. A blank 3D volume with the same dimensions as the template was then initialised, and each voxel corresponding to a coordinate was incremented by one to reflect any frequency of overlap of coordinates. Lastly, the density image was smoothed by a 6‐mm FWHM Gaussian kernel. Colour bar represents spatial density of S1 coordinates across participants (0–0.16), reflecting relative density of coordinates following smoothing, with warmer colours (e.g., yellow) indicating greater overlap.

Left S1 was localised relative to the left M1‐FDI hotspot, estimated in BrainSight Neuronavigation by moving the left M1‐FDI scalp coordinates 20 mm along the X axis and −5 mm along the Y axis. This protocol was made in consideration of a systematic review finding that S1 lies approximately 2 cm lateral and 0.5 cm posterior to the FDI motor hotspot (Holmes et al. 2019; Holmes and Tamè 2019). The coil handle was held at a 90° angle relative to the sagittal plane, as this has been shown to be an effective orientation to maximise S1 stimulation and minimise unwanted M1 responses (Tamè and Holmes 2023). Across both experiments, the average S1 MNI coordinates were −46.47, −16.36, 54.1 (SD = 2.58, 5.6, 2.82; *x*, *y*, *z*; Figure 1).

#### 2.3.3 Sham and Control Sites

**FIGURE 2:**
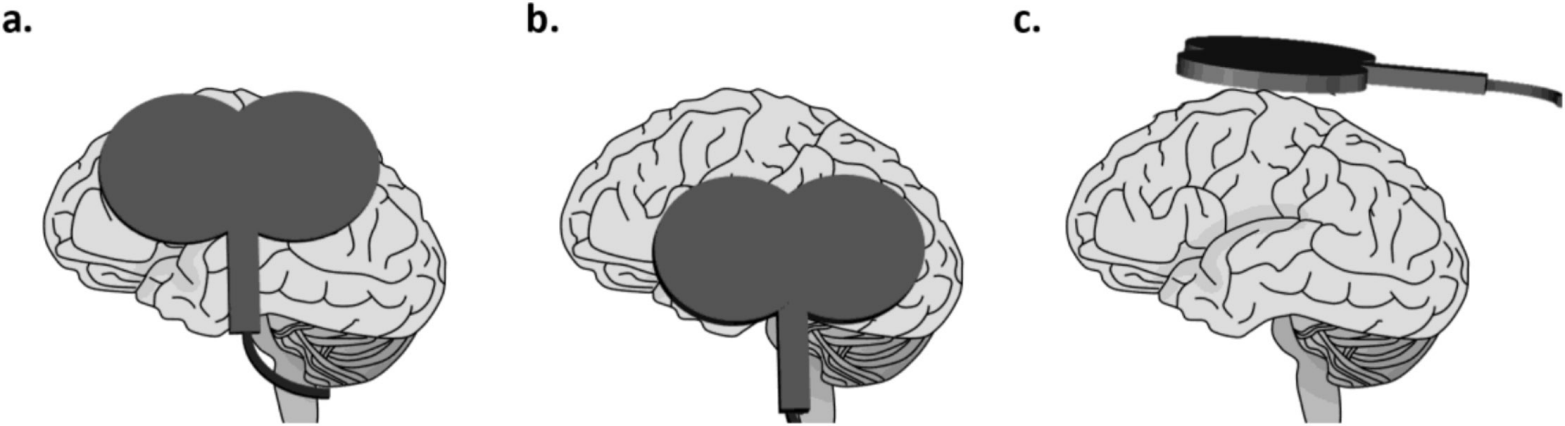
TMS conditions and coil location and orientation over the scalp. (a) Experimental target primary somatosensory cortex (S1), (b) active control inferior parietal lobe (IPL) and (c) sham vertex with a flipped coil directed away from the scalp.

In the sham condition, the coil was held over the vertex with a handle position of 180° relative to the sagittal plane, and the coil was flipped with the active side facing upwards so that the magnetic field was dissipated into the air (Figure 2c). The left IPL acted as the active control site using the average MNI coordinates (−56, −56.1, 36.2) from Tamè and Holmes (2016). Although stimulation of this site produces TMS‐induced artefacts comparable to S1, a previous study showed IPL did not elicit any significant fMRI activation during tactile detection (Tamè and Holmes 2016). The coil handle was positioned at a 90° angle relative to the sagittal plane. However, coil orientation was adjusted at the start of the main experimental task to match the level of freely reported TMS‐related discomfort between S1 and IPL. On average, this resulted in a slight forward tilt of the coil, lifting the lower edge away from the scalp to minimise discomfort induced by facial or scalp muscle contractions.

## 3 General Procedure

### 3.1 Experimental Task

**FIGURE 3:**
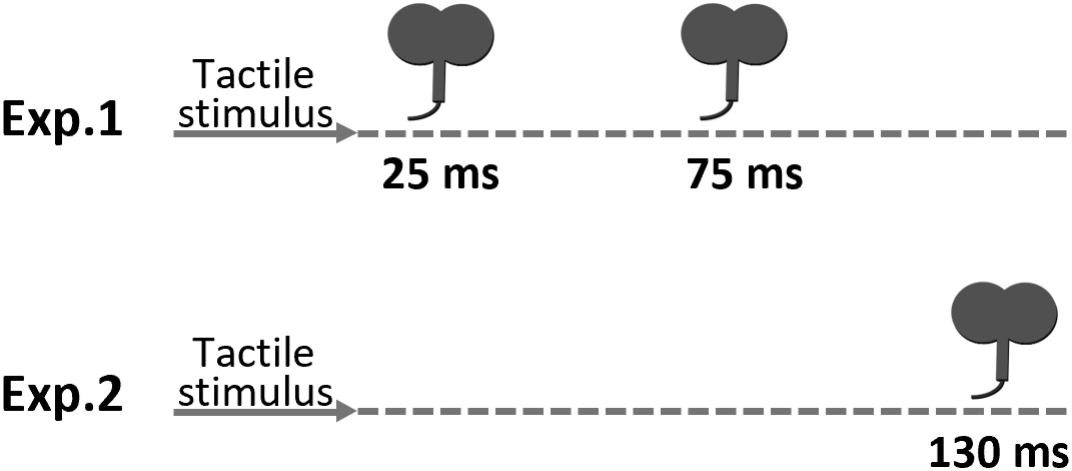
Schematic depiction of TMS pulse(s) timing relative to tactile onset in Experiments 1 and 2.

After completing TMS thresholding and localisation, followed by the tactile thresholding procedure, all participants completed a tactile detection Go/No‐go task. Participants sat with their right hand and forearm resting on a desk and fixed their gaze on a white fixation cross centrally positioned on a monitor 60 cm from the participant. The task consisted of six blocks of 20 trials. At the start of a trial, the fixation cross turned black for 4 s, after which the cross turned back to white to indicate the end of a trial. The inter‐trial interval (ITI) was 2.5 s in order to prevent any carryover effects of tactile stimulation or spTMS from the previous trial. A tactile stimulus was pseudorandomly presented on 50% of trials (10/20); a 0‐mA stimulus intensity was delivered on trials with no tactile stimulus. Tactile stimulus presentation occurred at a randomly jittered timepoint between 1 and 2 s from the start of the trial. Participants were instructed that on every trial, they may or may not feel a stimulation on the index finger and to press the ‘0’ key with the left index finger when they felt a stimulus. The left index finger was rested on the ‘0’ key throughout. No response was required if no stimulus was perceived. spTMS was delivered on every trial at 120% of the M1‐FDI RMT. The timepoint of TMS stimulation varied across experiments (Figure 3). The location of TMS (S1, control or sham) was counterbalanced across blocks under an ABCCBA counterbalanced sequence, to which participants were randomly assigned. At the end of each block, participants rated distractibility of the TMS: ‘How distracting was the TMS from the task?’ on a scale from 1 (‘*Not distracting at all*’) to 10 (‘*Most distracting thing imaginable in this context*’). Two‐minute breaks were taken between experimental blocks to allow coil re‐positioning and participant rest (total time to complete the task was about 25 min). No feedback on accuracy was provided.

## 4 Analysis

Data are openly available on OSF (https://osf.io/avmep/). Task performance was quantified using signal detection analysis, whereby Hits were defined as ‘yes’ responses on target‐present trials, and False Alarms as ‘yes’ responses on target‐absent trials. Hits and False Alarms were used to determine tactile sensitivity (*d′*), calculated as *d′* = *z* (Hits) − *z* (False Alarms). Response bias was determined using the criterion (*c*), calculated as *c* = −0.5 × [*z* (Hits) + *z* (False Alarms)]. To enable *z*‐score transformations, a continuity correction was applied such that hit and false alarm rates of 0 and 1 were adjusted to 0.01 and 0.99, respectively.

For each experiment, paired‐samples *t*‐tests were used to compare sensitivity (*d′*) and criterion (*c*) across TMS conditions (SI, IPL, sham vertex). Supplementary analyses were conducted to assess potential non‐specific effects of TMS, including subjective TMS‐related distraction ratings, averaged across blocks for each stimulation site and analysed using paired‐samples *t*‐tests. Reaction time (RT; ms) was also examined as a secondary index of non‐specific effects on general response processes such as motor execution, calculated from ‘hits’ on stimulus‐present trials (‘go‐trials’) and averaged across the two blocks for each TMS site.

## 5 Experiment 1

Experiment 1 investigated the effect of double spTMS over contralateral S1 on tactile detection compared with sham and control TMS. spTMS was delivered 25 and 75 ms after tactile onset. Two pulses were chosen to improve the precision of targeting the tactile trace arising in S1 following afferent delivery, given that signal arrival time by median nerve stimulation is thought to be around 20 ms (Allison et al. 1991) but has also been associated with perceptual detection around 70 ms after touch (Palva et al. 2005). Furthermore, note that the effect of single or double pulse TMS has not been found to differently affect tactile detection performance (Tamè and Holmes 2016).

### 5.1 Results

**FIGURE 4:**
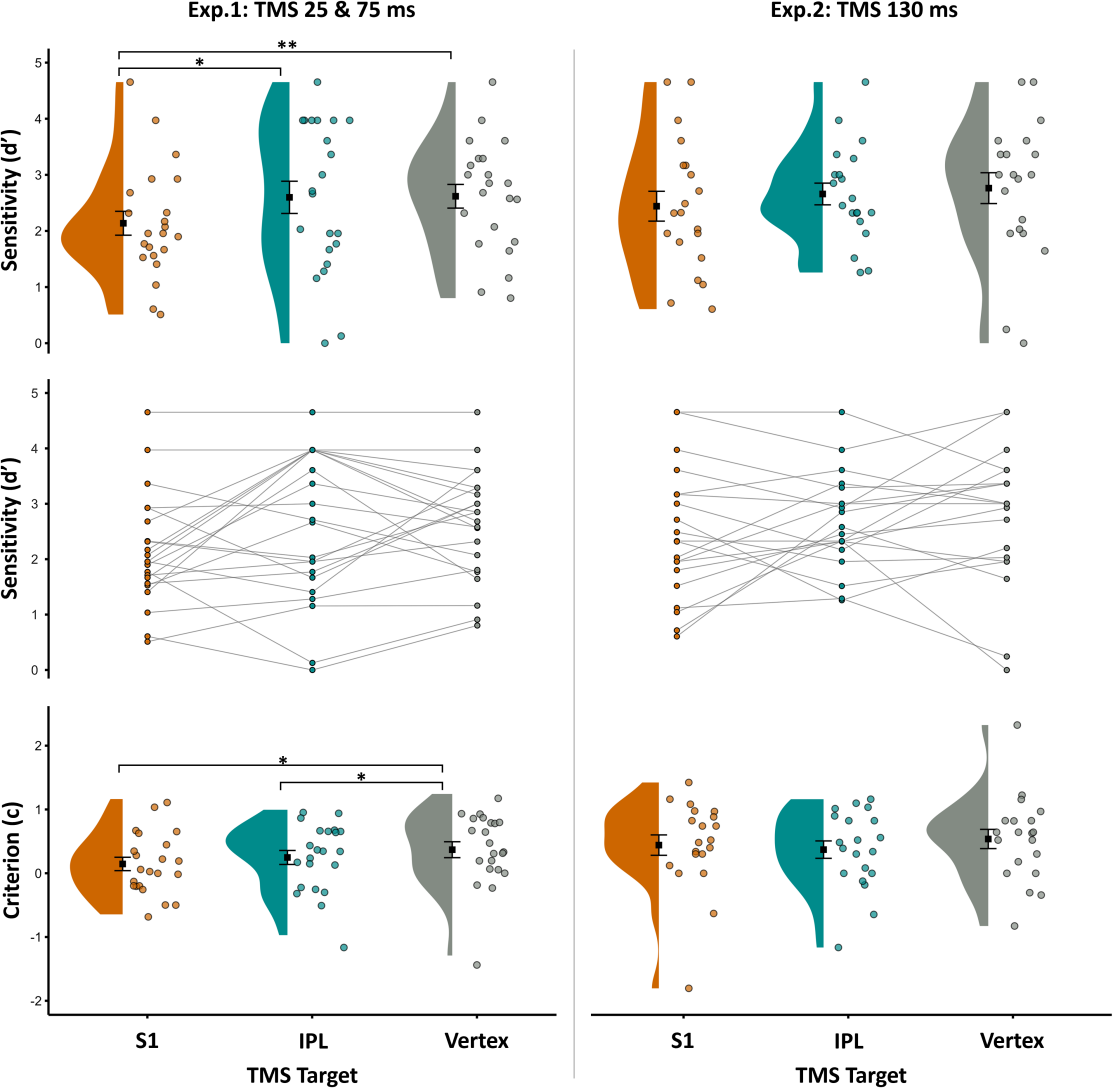
Sensitivity (*d*′) and response criterion (*c*) as a function of TMS target when early TMS was delivered after afferent onset (25 and 75 ms, Experiment 1) or late TMS delivery (130 ms, Experiment 2). Black squares show the mean and the standard error means (SEM). Half violins show data distribution, with overlaid jittered points showing individual participant values. The middle row displays within‐participant changes in sensitivity (*d*′) across TMS targets (spaghetti plot) *p ≤ 0.05, **p ≤ 0.01.

As shown in Figure 4 (left panel), one‐tailed paired sample *t*‐tests revealed tactile sensitivity (*d′*) was significantly reduced when TMS was delivered to S1 (M ± SE *d′* = 2.14 ± 0.21) compared with IPL (M ± SE *d′* = 2.6 ± 0.29; *t*(21) = −1.88, *p* = 0.037, *dz* = −0.38) and sham TMS (M ± SE *d′* = 2.62 ± 0.21; *t*(21) = −2.67, *p* = 0.007, *dz* = −0.48). Differently, sensitivity was comparable between IPL and sham TMS (*t*(21) = −0.09, *p* = 0.931, *dz* = −0.02). Criterion did not significantly differ when TMS was delivered to S1 (M ± SE *c* = 0.15 ± 0.11) compared with IPL (M ± SE *c* = 0.25 ± 0.11; *t*(21) = −0.88, *p* = 0.391, *dz* = −0.2). However, the criterion at S1 just reached a significant difference when compared with sham TMS (M ± SE *c* = 0.37 ± 0.13; *t*(21) = −2.07, *p* = 0.051, *dz* = −0.41) and the criterion was significantly lower at IPL than sham TMS (*t*(21) = −2.12, *p* = 0.046, *dz* = −0.21; Figure 4).

Self‐reported distraction was significantly higher at S1 (M ± SE = 4.76 ± 0.35) than at IPL (M ± SE = 4.22 ± 0.34; *t*(21) = 2.74, *p* = 0.012, *dz* = 0.34). Differently, distraction at vertex (M ± SE *d′* = 2.66 ± 0.23) was significantly lower than both S1 (*t*(21) = 7.31, *p* = < 0.001, *d* = 1.44) and IPL (*t*(21) = 6.14, *p* = < 0.001, *dz* = 1.07. RT did not significantly differ between S1 (M ± SE RT = 808.03 ± 47.64 ms) and IPL (M ± SE RT = 802.95 ± 45.87 ms; *t*(21) = 0.13, *p* = 0.896, *dz* = 0.02). However, RTs at vertex (M ± SE RT = 731.71 ± 46.02 ms) were significantly lower than at S1 (*t*(21) = 2.55, *p* = 0.019, *d* = 0.35) and at IPL (*t*(21) = 2.18, *p* = 0.040, *dz* = 0.33). Distraction and RT graphs can be found in Figure S1.

## 6 Experiment 2

Experiment 1 investigated the effect of early spTMS over contralateral S1 on tactile detection compared with sham and control TMS. However, the timeline of this contribution is unclear. Previous studies show that S1 activity persists at least 60 ms after tactile offset (Allison et al. 1992; Mauguière et al. 1997), and its signal recovery time has been reported to be around 110 ms (Hamada et al. 2002). Moreover, late (> 100 ms) activity in the somatosensory cortices has been associated with tactile detection performance (Schubert et al. 2006). Therefore, to investigate any ongoing contribution of S1 to perceptual detection beyond the tested critical window (< 100 ms), in Experiment 2, spTMS was delivered 130 ms after tactile onset.

### 6.1 Results

As shown in Figure 4 (right panel), one‐tailed paired sample *t*‐tests revealed that tactile sensitivity did not significantly differ when TMS was delivered over S1 (M ± SE *d′* = 2.44 ± 0.27) compared with IPL (M ± SE *d′* = 2.66 ± 0.19; *t*(19) = −0.98, *p* = 0.169, *dz* = −0.2) and, when compared with sham TMS (M ± SE *d′* = 2.76 ± 0.28; *t*(19) = −1.16, *p* = 0.131, *dz* = −0.27). Sensitivity was also comparable between the active control and sham sites; IPL and sham TMS (*t*(19) = −0.46, *p* = 0.648, *dz* = −0.09). Criterion did not significantly differ when TMS was delivered over S1 (M ± SE *c* = 0.44 ± 0.16) compared with IPL (M ± SE *c* = 0.37 ± 0.14; *t*(19) = 0.76, *p* = 0.459, *dz* = 0.01) nor sham TMS (M ± SE *c* = 0.54 ± 0.15; *t*(19) = −0.74, *p* = 0.471, *dz* = −0.14). Furthermore, the criterion did not differ between IPL and sham TMS (*t*(19) = −1.68, *p* = 0.110, *dz* = −0.26).

Self‐reported TMS distraction for each TMS site was averaged across the two blocks of stimulation, as in Experiment 1. Distraction did not significantly differ between S1 (M ± SE = 3.78 ± 0.29) and IPL (M ± SE = 3.53 ± 0.37; *t*(19) = 0.89, *p* = 0.387, *dz* = 0.16). However, distraction was significantly lower for sham TMS (M ± SE = 2.2 ± 0.27) compared with TMS over either S1 (*t*(19) = 7.53, *p* = < 0.001, *dz* = 1.25) and IPL (*t*(19) = 3.87, *p* = < 0.001, *dz* = 0.90. Lastly, RT did not significantly differ between S1 (M ± SE RT = 714.57 ± 52.71 ms) and IPL (M ± SE RT = 689.07 ± 40.59 ms; *t*(19) = 0.70, *p* = 0.493, *dz* = 0.12) nor S1 and sham TMS (M ± SE RT = 642.39 ± 55.36 ms; *t*(19) = 1.79, *p* = 0.089, *d* = 0.3) and did not differ between IPL and sham (*t*(19) = 1.49, *p* = 0.153, *dz* = 0.19).

## 7 Discussion

This study investigated the temporal dynamics of primary somatosensory cortex involvement in tactile detection using inhibitory spTMS. Tactile sensitivity was significantly reduced when TMS was applied over the contralateral S1 shortly after tactile stimulus onset (25 and 75 ms; Experiment 1), but not when applied at a later time (130 ms; Experiment 2). This effect was specific to S1, as no comparable reduction in sensitivity was observed when spTMS was applied over an active control site (IPL) or using sham TMS (over the vertex with a flipped coil). These findings strongly support the notion that S1 plays a critical, yet temporally constrained, role in conscious tactile stimulus detection, reflecting its key role in the initial encoding.

Our findings of early but not late S1 involvement in tactile detection stand somewhat in contrast to M/EEG research, which has demonstrated that late S1 activity is associated with conscious detection. Specifically, the P100 and N140 components have been shown to correlate with conscious stimulus detection (Auksztulewicz et al. 2012; Forschack et al. 2020; Schröder et al. 2021; Schubert et al. 2006; Wühle et al. 2011), suggesting that late S1 activity contributes to detection. To reconcile these findings with the present results, we propose that the early causal contribution of S1 reflects its role in the initial encoding of the tactile signal. This in turn provides a neural representation of touch that can be immediately or later distributed across cortical networks to support perceptual functions such as tactile detection, discrimination, retention and learning. Accordingly, disruption of the signal during this early time window may impair both immediate and later processing stages that depend on this representation. Supporting this view, previous studies have shown that TMS over contralateral S1 disrupts performance in tasks of vibrotactile frequency discrimination and tactile working memory (Harris et al. 2002; Morley et al. 2007), as well as temporal discrimination tasks (Hannula et al. 2008).

Moreover, we suggest that the temporal specificity of S1 involvement observed in our study reflects a distributed neural architecture underlying tactile processing that extends beyond S1. Within such a framework, early S1 activity supports feedforward encoding of the tactile stimulus, rendering S1 necessary, but not in itself sufficient, for conscious detection. This interpretation helps reconcile why early somatosensory components in M/EEG do not consistently correlate with conscious perception, despite the early causal role of S1 demonstrated here. Indeed, early somatosensory components such as the P50, P60 and N80 are often observed for both detected and undetected stimuli (Schubert et al. 2006) and appear to reflect stimulus properties such as intensity rather than perceptual awareness (Forschack et al. 2020; Förster et al. 2025; Schröder et al. 2021). In contrast, later components such as the P100 and N140 more reliably track detection and detection probability, suggesting that conscious perception depends on, or is reflected in, subsequent integrative processing beyond early sensory encoding (Pereira et al. 2025).

By a subsequent integrative processing beyond S1, we refer to the distributed network of cortical and subcortical regions that likely support conscious tactile awareness, encompassing the secondary somatosensory cortex (S2), posterior parietal cortex (PPC) and subcortical structures such as the thalamus. For example, source localisation studies indicate that later components such as the P100 arise not only from S1 but also from bilateral S2 and parietal regions (Auksztulewicz et al. 2012; Auksztulewicz and Blankenburg 2013). Complementing this, recent intracranial recordings in patients undergoing neurosurgery demonstrated that neuronal activity in subcortical structures, including the thalamus and subthalamic nucleus, correlated with conscious detection at latencies of around 150 and 300 ms (Pereira et al. 2025), highlighting the contribution of cortico‐subcortical circuits. These findings align with the seminal work performed in non‐human primates, showing that early sensory cortex primarily encodes stimulus features, whereas perceptual awareness emerges through subsequent, distributed processing in higher‐order regions, including S2, PPC, and the prefrontal cortex (de Lafuente and Romo 2005, 2006).

Within this framework, disrupting early S1 may impair the formation of an adequate sensory representation, thereby reducing the likelihood that the stimulus is successfully propagated across the necessary networks supporting conscious detection. This interpretation is consistent with evidence that the contribution of S1 to tactile detection in experimental contexts depends on the set task demands (Tamè and Holmes 2016), suggesting network‐level engagement underlying tactile perception beyond S1. Moreover, neural processing underlying conscious stimulus detection may also vary as a function of the nature of the stimulus. Indeed, recent event‐evoked potential (ERP) evidence demonstrates distinction in the neural processes underlying electrical and mechanical tactile detection, with differential contributions of early and late somatosensory components (Förster et al. 2025). This suggests that the neural pathways supporting tactile awareness are not fixed but instead reflect a broader and more flexible processing architecture.

The observed early involvement of S1 in tactile detection aligns with previous TMS investigations showing that TMS over the contralateral somatosensory cortices impairs detection when applied 20–50 ms after electrotactile stimulation (Cohen et al. 1991; Hannula et al. 2005; McKay et al. 2003; Seyal et al. 1992). However, unlike earlier work, the present study controlled for non‐specific TMS effects such as auditory and somatosensory artefacts by including an active control site, consistent with recent methods used by Tamè and Holmes (2016). Given that higher TMS intensity can increase tactile suppression (McKay et al. 2003), a finding possibly attributable to non‐specific TMS effects, the current study also collected subjective TMS‐related distraction ratings. In Experiment 1, expectedly, S1 and IPL were rated as more distracting than sham TMS. However, unexpectedly, S1 was rated as significantly more distracting than IPL. Despite this, the reduction in tactile sensitivity following S1 TMS cannot be attributed solely to distraction. If distraction inherently impaired tactile sensitivity, IPL TMS should also have led to reduced sensitivity compared with sham, given that it was also rated as more distracting than sham. Instead, sensitivity was comparable between IPL and sham conditions. We propose that the higher distraction ratings for S1 may reflect a post hoc attribution of task difficulty, where participants' distraction ratings were influenced by perceived decreased performance. This interpretation is further supported by Experiment 2, in which no differences in either sensitivity or distraction ratings were observed between S1 and IPL.

Our findings on the key role of S1 in tactile sensitivity align with those of Tamè and Holmes (2016), who reported reduced vibrotactile sensitivity when TMS was applied over S1 25 and 75 ms post‐stimulus onset during a tactile yes/no task. However, differently to our results, which showed that S1 and IPL TMS led to a lower response criterion compared with sham, Tamè and Holmes (2016) found a higher criterion (more conservative responding) following S1 and IPL TMS. We note, however, that this discrepancy may be partly explained by methodological differences. Their study used a no‐TMS condition with the coil held away from the head; contrastingly, we administered sham TMS with a rotated coil held on the scalp over the vertex.

The absence of significant differences in criterion across TMS sites in Experiment 2 may be explained by the longer inter‐stimulus interval (130 ms), occurring outside of the critical window for tactile detection. At this later stage, perceptual decisions may already be committed, making participants less susceptible to shifts in response strategy. This further supports the idea that S1 involvement in tactile detection is temporally constrained.

S1 lies in close anatomical proximity to the primary motor cortex (M1), and the two regions are densely interconnected, with tactile input playing a critical role in motor control and in guiding motor behaviour (Johansson and Flanagan 2009). In TMS experiments, it is therefore always possible that such effects of S1 stimulation might further reflect, to an extent, indirect influences on M1 arising from either TMS current spread or functional connectivity. However, in our study, notable aspects of the data argue against a purely motor‐based account. Notably, despite the significant reduction in tactile sensitivity, RTs never differed between S1 and the active control site. If motor cortex stimulation were driving the effect, it is reasonable to expect corresponding changes in response speed given its implication in action planning and motor execution. Moreover, the response criterion did not differ between S1 and active control TMS; however, if indirect stimulation of M1 were to influence perceivability of the sensory signal, it would be reasonable to expect changes in response strategy in line with increased task difficulty, including more conservative responding. Together, these findings suggest that the observed reduction in sensitivity reflects genuine changes in perceptual processing.

Nonetheless, the close functional relationship between S1 and M1 could reflect a network‐level contribution to tactile detection. S1 projections to M1 are known to be predominantly inhibitory (Widener and Cheney 1997), and a single peripheral tactile input has been shown to suppress TMS‐induced MEPs (Chen et al. 1999). Within this framework, disruption of S1 may alter downstream processing in this sensorimotor network. Interestingly, however, if the observed effects of the present study were primarily driven by tactile M1 inhibition, similar effects on sensitivity would be expected in Experiment 2, given that modulatory influences on M1 can persist for several hundred milliseconds (Turco et al. 2018). The absence of effects with TMS delivered at 130 ms in Experiment 2, therefore, suggests that the observed changes in sensitivity are attributable to the disruption of early somatosensory processing rather than motor cortex involvement per se. It is, however, evident that future research would benefit from aiming to disentangle the respective contributions of S1 and M1 to tactile detection, particularly within the context of their functional interactions.

A potential limitation of the present study is the use of different TMS protocols across experiments, with double‐pulse TMS in Experiment 1 and spTMS in Experiment 2. This difference may constrain direct comparisons between early and late effects, given that variation in pulse numbers could, in principle, influence the magnitude of disruption. In addition, the use of separate participant groups can limit the ability to make direct within‐subject comparisons of early and late stimulation effects, thereby constraining conclusive inferences regarding temporal differences. However, these concerns are mitigated by the fact that all key comparisons were made within experiments using control and sham TMS conditions matched for stimulation parameters. Moreover, prior work has shown comparable effects of single‐ and double‐pulse TMS on tactile detection (Tamè and Holmes 2016), and task difficulty was controlled across participants in both experiments using a standardised adaptive staircase procedure.

A further factor to consider, common to all TMS studies, is the spatial precision of TMS. Although S1 was localised for each participant using functionally defined M1 FDI hotspots and was based on recent MRI and fMRI‐guided meta‐analytic and experimental procedures (Holmes et al. 2019; Holmes and Tamè 2019; Tamè and Holmes 2023), this approach can introduce variability in the accuracy of targeting. Future studies could be strengthened by using individualised MRI‐guided neuronavigation to optimise within‐participant spatial precision. Importantly, these limitations do not detract from the central finding that the transient disruption of S1 within an early post‐stimulus window (< 100 ms) selectively impairs tactile sensitivity relative to both an active control site and sham stimulation, with no corresponding effect observed when a later time point was investigated.

It is also interesting to acknowledge the artificial nature of the tactile stimulation used in the present study, as is common across the vast majority of experimental paradigms in this domain. Specifically, electrotactile stimulation, while offering high temporal control and reliable threshold calibration, does not fully replicate the spatiotemporal properties of naturalistic touch. Accordingly, the findings should be interpreted within the context of a controlled sensory paradigm. Future research should seek methods capable of probing more naturalistic forms of tactile experiences, thereby improving generalisability to everyday tactile perception and its underlying neural processes.

Overall, our results demonstrate that tactile detection arises from temporally staged involvement of S1. Importantly, we demonstrate that the effect of S1 TMS is specific to perceptual sensitivity rather than due to non‐specific TMS effects. Within the context of the current methodology, this supports a causal role for S1 in tactile detection, which we propose arises from disrupted initial encoding. This disruption likely reduces the probability that the stimulus reaches higher‐level stages and perceptual awareness.

## Author Contributions

**Louisa Gwynne:** conceptualization, data curation, formal analysis, investigation, methodology, project administration, validation, visualization, writing – original draft, writing – review and editing. **Luigi Tamè:** conceptualization, data curation, formal analysis, methodology, project administration, resources, software, supervision, validation, writing – review and editing.

## Funding

This work was supported by the Economic and Social Research Council, ES/P00072X/1.

## Conflicts of Interest

The authors declare no conflicts of interest.

## Supporting information


**Figure S1:** Tactile detection reaction times and TMS distraction ratings as a function of TMS target when early TMS was delivered after afferent onset (25 and 75 ms, Experiment 1) or late TMS delivery (130 ms, Experiment 2). Reaction times are given for ‘hits’ (a ‘go’ response on target‐present trials). Black squares show the mean and standard error means (SEM). Half violins show data distribution, and coloured circles are jittered individual participant data points. *p ≤ 0.05, **p ≤ 0.01, ***p ≤ 0.001.

## Data Availability

Data are openly available on the Open Science Framework (OSF) at https://osf.io/avmep/. Analysis scripts are available upon request to the corresponding authors (L.G. or L.T.).
