# Supplementary material for: Conscious Tactile Perception Depends on Early Processing in Primary Somatosensory Cortex
Source: Eur J Neurosci. 2026 May 15;63:e70548. doi: 10.1111/ejn.70548 (PMC13179477; doi:10.1111/ejn.70548)
Supplement: Supplementary file 1 — Figure S1: Tactile detection reaction times and TMS distraction ratings as a function of TMS target when early TMS was delivered after afferent onset (25 and 75 ms, Experiment 1) or late TMS delivery (130 ms, Experiment 2). Reaction times are given for ‘hits’ (a ‘go’ response on target‐present trials). Black squares show the mean and standard error means (SEM). Half violins show data distribution, and coloured circles are jittered individual participant data points. *p ≤ 0.05, **p ≤ 0.01, ***p ≤ 0.001. [file EJN-63-0-s001.docx]

**
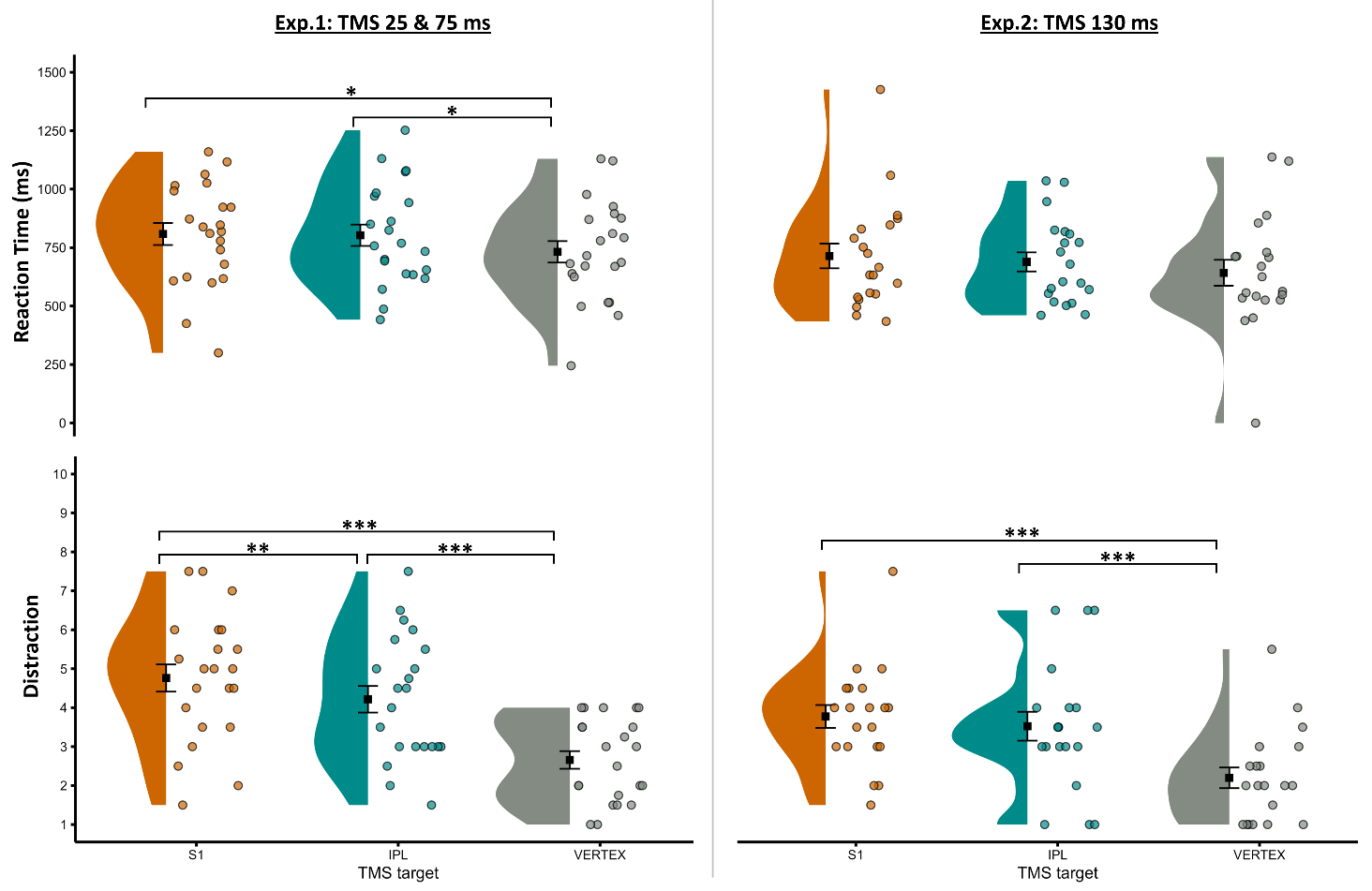
**

**Figure S1*.*** Tactile detection reaction times and TMS distraction ratings as a function of TMS target when early TMS was delivered after afferent onset (25 & 75 ms, Experiment 1) or, late TMS delivery (130 ms, Experiment 2). Reaction times are given for “hits” (a “go” response on target-present trials). Black squares show mean and standard error means (SEM). Half violins show data distribution, coloured circles are jittered individual participant data points**P* ≤ .05, ***P* ≤ .01, ****P* ≤ .001
